# Botulinum Toxin A Injection in the Bladder Neck: A Promising Treatment for Urinary Retention

**DOI:** 10.1155/2016/6385276

**Published:** 2016-03-10

**Authors:** Marianne Alam, Joseph Zgheib, Mohamad-Fadi Dalati, Fouad El Khoury

**Affiliations:** ^1^University of Balamand, St. George Hospital University Medical Center (SGHUMC), Beirut 1100 2807, Lebanon; ^2^Collaborateur Scientifique d'Urologie, CHU St. Pierre, 1000 Brussels, Belgium

## Abstract

Secondary to failure of optimal medical therapy and the high morbidity that accompanies surgical techniques in high risk patients, the use of de novo treatments including botulinum toxin A is emerging in the treatment of benign prostatic hyperplasia (BPH). However, the treatment of urinary retention secondary to BPH via injecting botulinum toxin into the bladder neck is not well established in the literature. This case report describes the case of a 75-year-old male patient with a chronic history of obstructive lower urinary tract symptoms (LUTS) and multiple comorbidities who was admitted to the hospital for management of recurrent urinary retention. The patient was not a surgical candidate for transurethral incision of the prostate (TUIP) or transurethral resection of the prostate (TURP). Botulinum toxin injection into the bladder neck was performed with very satisfying results. Botulinum toxin injection in the bladder neck presents a promising minimally invasive, tolerated, and cost-effective approach for the treatment of urinary retention in patients with benign prostatic obstruction who are not candidates for surgery or in whom medical treatment has failed. More research is needed to identify the efficacy of this novel approach.

## 1. Introduction

Benign prostatic hyperplasia (BPH) is a benign enlargement of the prostate gland that affects 30% of males between 50 and 60 years old and up to 90% of males above the age of 85 years [1]. Symptoms of BPH result from obstruction of the prostatic urethra and gradual failure of bladder function, which result in a deficient bladder emptying that can lead to complications, including acute urinary retention [2]. Although several treatment options are available, following the adverse effects of medical treatments and the morbidity that accompanies surgical techniques in high risk patients, the use of de novo treating modalities is emerging in the treatment of benign prostatic hyperplasia [3]. Yet, available research on this topic has mainly reported injections sites into the prostate. To the best of our knowledge, no study has described Botox injections into the bladder neck as an alternative treatment for BPH. The patient described in this case report underwent bladder neck injection for the management of urinary retention with satisfying postprocedure outcomes.

## 2. Case Presentation

A 75-year-old male patient known to have hypertension, diabetes mellitus for 35 years, chronic kidney disease (baseline creatinine 2.4 mg/dL), congestive heart failure (ejection fraction 37%), obstructive lower urinary tract symptoms (LUTS), and peripheral vascular disease presented for syncope. The list of the patient's medications is shown in Table 1. Investigations in the hospital were done and the patient was diagnosed with decompensation of heart failure by pneumonia. During his hospital stay, the patient developed acute urinary retention (postvoid residue PVR 1200 mL). Urologic evaluation revealed a chronic history of bladder outlet obstruction with an IPSS score of 32 despite treatment with alpha blockers. Digital rectal exam was compatible with a small and soft prostate. Cystoscopy was done and showed a mild bilobar prostatic hypertrophy but significantly obstructive and high bladder neck. The patient was scheduled for transurethral incision of the prostate (TUIP) but clearance for surgery was denied from both anesthesia and cardiology teams due to unfavorable cardiac status. Later on, the patient presented to the emergency room for acute urinary retention on several occasions. A 16 Fr hydrogel coated Foley catheter was kept for 16 days and 2 attempts to remove the catheter resulted in failure even after maximizing medical therapy for bladder outlet obstruction. An ultrasound was done for further evaluation revealing a 25-gram prostate. The patient was informed that a trial of onabotulinumtoxinA injection into the bladder neck can be attempted under local anesthesia. He was well informed that this treatment is only being done in clinical trials and accepted to undergo the procedure.

## 3. Treatment

**Figure 1:**
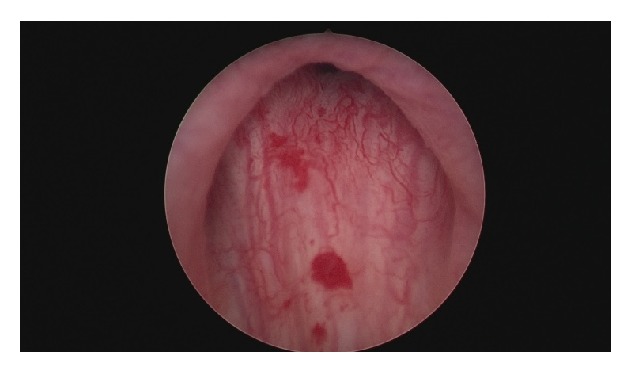
Bladder neck hypertrophy before the injection, causing almost complete bladder outlet obstruction (BOO).

**Figure 2 fig2:**
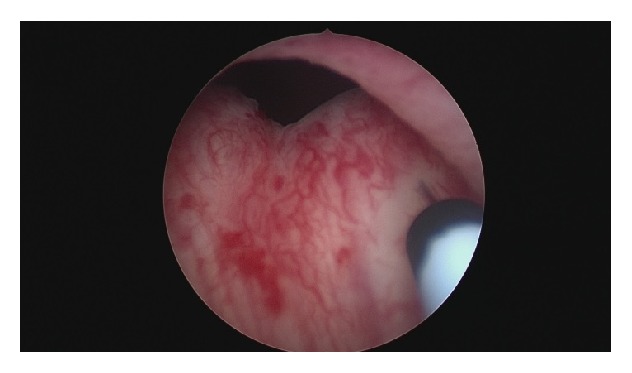
Bladder neck Botox injections at 3 o'clock.

**Figure 3:**
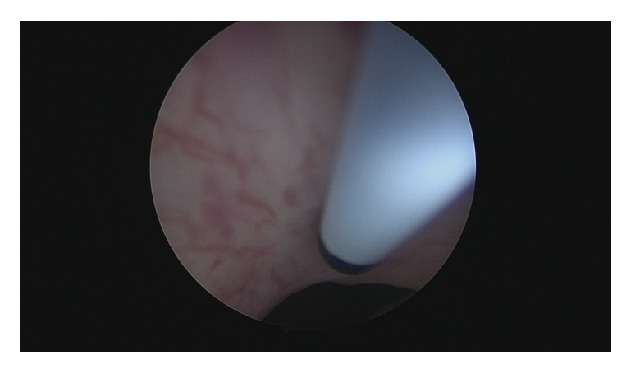
Botox injection over anterior bladder neck.

Under local anesthesia, inoculation of 100 U of onabotulinumtoxinA, BOTOX®, diluted in 10 cc of normal saline solution (divided into 10 injections of 1 cc each) into the bladder neck was performed at 3, 6, 9, and 12 o'clock (Figures 1 [Fig fig2]–3). The patient tolerated well the procedure and a Foley catheter was placed.

## 4. Outcome and Follow-Up

**Figure 4:**
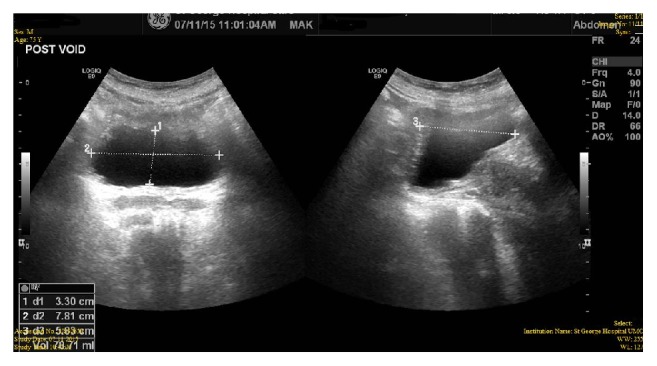
PVR after Botox injections: 78 mL.

The Foley catheter was removed day 1 post-op and the patient voided spontaneously. Postvoid residue was measured using suprapubic ultrasound which gave an estimation of 78 mL (Figure 4). The patient was discharged home. 30 days post-op, the patient was received in the clinics for a follow-up visit. He denied signs or symptoms of UTI, dysuria, hematuria, retention, or any other adverse event. His I-PSS score was noted to be 9, suggesting a significant objective improvement.

## 5. Discussion

Botulinum toxin type A has been used by neurologists as a treatment for neuromuscular conditions such as dystonia and spasticity and has recently been publicized for the management of facial wrinkles. The usefulness behind its use lies behind its property of inhibiting acetylcholine release at the neuromuscular junction. De novo uses of botulinum toxin have expanded to other conditions such as hypersecretory disorders, tics, tremor, stuttering, different pain syndromes, gastrointestinal smooth muscle/sphincter spasms, and many urological conditions, notably symptomatic treatment of overactive bladder (OAB) [4]. As recommended by the European Association of Urology, botulinum toxin injection in the detrusor muscle of the bladder is the most effective minimally invasive therapy to decrease neurogenic detrusor overactivity (Grade A recommendation) [5].

The intraprostatic injection of botulinum neurotoxin type A (BoNT-A) is a minimally invasive usage of the drug to treat benign prostatic hyperplasia (BPH) and subsequent lower urinary tract symptoms (LUTS). Previous studies have shown improvement of maximum urinary flow rate, quality-of-life index, and reduction of International Prostate Symptoms Score (IPSS), prostate-specific antigen (PSA) level, postvoid residual volume, and prostate volume [6]. However, unlike other studies that have focused on intraprostatic injection of BoNT-A, this case report which performed transurethral injection into the bladder neck, notably the anterior urethral wall, presents a promising and safe novel therapy for the treatment of urinary retention in patients with bladder neck hypertrophy who are poor surgical candidates or in whom medical treatment has failed. As our case have shown, after bladder neck injection, the patient presenting with history of recurrent episodes of urinary retention had major improvements in postvoid residue and IPSS scores. Besides, the use of transurethral approach provides a direct view of the bladder neck, median lobe, and transition zone and presents a secure method as compared to transrectal and transperineal injections [7]. Also, transurethral injection of BoNT-A was done with only local anesthesia compared to other studies using epidural and general or light general anesthesia [6]. This route of administration has been observed to be a safe procedure. Studies have revealed no early or late complications and patients usually tolerate the injections well [8]. The underlying mechanism behind the symptoms relief is related to volume shrinkage (decreasing the level of obstruction by the high bladder neck) as well as smooth muscle inhibition and relaxation [9].

The relief in symptoms is most probably related to the relaxing properties of the toxin as observed in other applications of the drug. Although not demonstrated, the dose of 100 U seems appropriate to obtain satisfactory results.

Multiple minimally invasive procedures that deal with medically resistant, surgically contraindicated BPH have emerged. One of the latter techniques “Urolift” is used to relieve urinary symptoms caused by BPH. It is a minimally invasive approach that lifts the enlarged prostate, relieving obstruction, and can be done under local anesthesia [10]. However, such technique, like others, deals mainly with BPH rather than high bladder neck presented in our case and may not be recommended for patients with high bladder neck or modest median lobe.

## 6. Conclusion

Wrapping up this case report, botulinum toxin injection into the bladder neck appears to offer a new promising treatment option for bladder neck hypertrophy. However, further trials are needed to establish the validity of this new indication as well as the adequate dosage of the toxin required. Establishment of follow-up protocols, time interval between injections, and comorbidities associated with this treatment need to be appropriately addressed. The ease of installation, the lack of need of anesthesia, the possibility of outpatient treatment models, and the efficacy of such minimally invasive approach to persistent LUTS due to bladder neck hypertrophy seem to be of a promising value. Our team will continue to further study such approach, with enrolment of new patients for better understanding of the benefits of such treatment modalities. To the best of our knowledge, such minimally invasive approach for treatment of medication-refractory urinary retention due to bladder neck hypertrophy is the first of its kind in the Middle East region.

## Figures and Tables

**Table 1:** Medications.

Medication	Dosage and frequency
Cynt 4 mg	1 tablet once daily
Isoptin 240 mg	1 tablet once daily
Aspicor 81 mg	1 tablet once daily
Silosin 8 mg	1 tablet once daily
Trajenta 5 mg	1 tablet once daily
